# CMA-DTI: a cross-modal fusion and attentive interaction network for interpretable drug-target interaction prediction

**DOI:** 10.3389/fbinf.2026.1861685

**Published:** 2026-06-11

**Authors:** Chi Qin, Denggao Zheng, Ziyang Wang, Yu Li, Jingrui Cao, Houchun Qiu, Hongxing Kan, Liping Sun, Yu Liu, Jili Hu

**Affiliations:** 1 School of Medical Information Engineering, Anhui University of Chinese Medicine, Hefei, China; 2 Center for Xin’an Medicine and Modernization of Traditional Chinese Medicine of IHM, Anhui University of Chinese Medicine, Hefei, China

**Keywords:** attention mechanism, deep learning, drug discovery, drug-target interaction prediction, interpretability, multi-modal learning

## Abstract

**Background:**

Drug–target interaction (DTI) prediction is an important task in early-stage drug discovery. Although deep learning methods have improved predictive performance, effectively integrating heterogeneous drug representations and providing interpretable evidence for local interaction patterns remain challenging.

**Methods:**

We propose CMA-DTI, a cross-modal fusion and attentive interaction framework for DTI prediction. CMA-DTI integrates GCN-based molecular graph representations, ChemBERTa-derived SMILES representations, and ESM-2 protein residue embeddings. An intra-drug cross-attention module models soft relevance patterns between graph nodes and SMILES tokens, while a drug–protein multi-head attention module captures local relevance between fused drug nodes and protein residues.

**Results:**

On BindingDB and BioSNAP, CMA-DTI achieved competitive performance compared with representative machine learning and deep learning baselines. Cold-drug and cold-target evaluations showed that the model retained predictive ability under unseen-drug and unseen-target settings. Ablation results indicated that intra-drug cross-attention performed better than concatenation, addition, and gated fusion. In a representative structural case, attention-ranked residues showed moderate enrichment in ligand-binding pocket residues compared with random rankings.

**Conclusion:**

CMA-DTI provides a practical framework for multimodal DTI prediction by combining graph structure, chemical language representations, and protein language model embeddings. Its attention patterns offer hypothesis-generating molecular relevance evidence, but should not be interpreted as causal mechanistic explanations.

## 1 Introduction

Accurately identifying drug–target interactions (DTIs) is an important task in drug discovery (Schneider et al., 2020). DTIs provide useful information for understanding drug mechanisms (Schenone et al., 2013), accelerating lead compound screening, and identifying potential off-target effects (Askr et al., 2023). However, experimental identification of DTIs is time-consuming and costly, making it difficult to apply at large scale. Therefore, computational methods for DTI prediction have become an important complement to experimental screening.

Early computational methods were mainly based on structure-based or ligand-based strategies (Hasan et al., 2022; Jiménez-Luna et al., 2021). Structure-based approaches, such as molecular docking, depend on the availability and quality of three-dimensional protein structures, which limits their application to proteins without resolved structures (Berenger et al., 2021; Abdul Raheem and Dhannoon, 2024). Ligand-based methods infer interactions from known active molecules, but their performance can decrease when applied to novel targets or sparse interaction data (Yu et al., 2023). These limitations motivate the development of data-driven methods that can learn representations from large-scale DTI datasets.

Deep learning has been increasingly applied to DTI prediction because it can learn feature representations directly from molecular and protein data (Bian et al., 2023; Paul et al., 2021; Ren et al., 2023; Vamathevan et al., 2019). Early models used inputs such as molecular fingerprints and amino acid compositions (Rogers and Hahn, 2010). Later methods introduced more expressive neural architectures. For example, DeepConv-DTI (Lee et al., 2019) used convolutional neural networks to extract local protein sequence patterns, whereas GraphDTA (Nguyen et al., 2021) used graph neural networks to encode drug molecular structures. Attention-based models such as MolTrans (Huang et al., 2021) and DrugBAN (Bai et al., 2023) further modeled local interaction patterns between drugs and targets. More recent methods have explored advanced interaction and fusion modules. For example, BINDTI (Peng et al., 2025) combines an ACmix protein encoder with a bi-directional intention network, and MGMA-DTI (Li et al., 2025) uses multi-order gated convolutions and multi-attention fusion to extract interaction features. These methods generally formulate DTI prediction as a binary classification task, in which drug and protein representations are encoded and then mapped to an interaction probability.

Despite these advances, several challenges remain in deep learning-based DTI prediction. First, drug molecules can be represented from complementary views, including molecular graphs and SMILES sequences. Molecular graphs encode atom connectivity and local topology, whereas SMILES-based chemical language representations capture contextual chemical patterns. However, many existing methods either rely on a single drug representation or combine multiple modalities through simple concatenation, which may not fully model the relationship between graph-level atom representations and SMILES-level chemical tokens. Second, although attention mechanisms are increasingly used in DTI models, attention maps are often presented only as qualitative visualizations. More quantitative analyses are needed to assess whether attention-ranked atoms or residues are associated with experimentally observed binding regions.

Pre-trained language models provide useful representations for both proteins and molecules. ESM-2 (Lin et al., 2023) generates residue-level protein embeddings with evolutionary and structural information, while ChemBERTa (Ahmad et al., 2022) captures chemical language patterns from SMILES strings. Recent methods such as TriDTI (Yun et al., 2026), BioT5 (Pei et al., 2023), and SiamDTI (Zhang et al., 2024) have explored multimodal or pre-trained representations for molecular and protein interaction tasks. These studies indicate the value of combining graph, sequence, and language-model-derived features. However, how to integrate graph-based drug topology with SMILES-derived chemical representations and connect the fused drug features with protein residue representations remains an important problem.

To clarify the methodological position of CMA-DTI relative to representative attention-based and multimodal DTI models, Table 1 summarizes their main methodological characteristics in representation learning, interaction modeling, intra-drug fusion, and interpretability evidence. Unlike methods that mainly apply attention at the drug-protein interaction level or perform general multimodal alignment, CMA-DTI introduces intra-drug cross-attention to model soft relevance between GCN-based graph nodes and ChemBERTa-derived SMILES tokens before drug-protein interaction modeling.

**TABLE 1:** Methodological comparison of representative attention-based and multimodal DTI models.

Method	Representation	Interaction/Fusion strategy	Interpretability evidence
MolTrans (Huang et al., 2021)	Drug/protein tokens	Transformer-based interaction	Attention visualization
DrugBAN (Bai et al., 2023)	Molecular graph + protein sequence	Bilinear attention	Attention visualization
MCANet (Bian et al., 2023)	Sequence features	Multi-head cross-attention	Not emphasized
MGMA-DTI (Li et al., 2025)	Drug graph + protein sequence	Gated conv. + multi-attention	Not emphasized
TriDTI (Yun et al., 2026)	Multimodal drug/target features	Cross-modal alignment + attention	Not emphasized
CMA-DTI	GCN nodes + ChemBERTa + ESM-2	Graph–SMILES + drug–protein attention	Pocket-level enrichment

This table summarizes methodological characteristics rather than direct performance ranking.

To address these issues, we propose CMA-DTI, a cross-modal fusion and attentive interaction framework for DTI prediction. CMA-DTI uses a Graph Convolutional Network to encode molecular graphs, ChemBERTa to encode SMILES sequences, and ESM-2 to encode protein sequences. An intra-drug cross-attention module is used to model soft relevance patterns between graph node embeddings and SMILES token embeddings. The resulting fused drug node representations are then combined with ESM-2 residue embeddings through a drug–protein multi-head attention module for interaction prediction.

The main contributions of this work are summarized as follows.

- Multimodal representation learning for DTI prediction. CMA-DTI integrates graph-based molecular representations, SMILES-derived chemical language representations, and protein language model embeddings to provide complementary information for DTI prediction.
- Soft graph–SMILES fusion. CMA-DTI introduces an intra-drug cross-attention module to model soft relevance patterns between GCN-based graph nodes and ChemBERTa-derived SMILES tokens, without assuming a deterministic one-to-one atom-token correspondence.
- Attention-based drug–protein interaction modeling. CMA-DTI uses a drug–protein multi-head attention module to model local relevance patterns between fused drug node representations and protein residue embeddings.
- Evaluation of generalization and attention-based interpretation. CMA-DTI is evaluated using benchmark comparisons, cold-drug and cold-target splits, fusion-strategy ablation, and quantitative pocket-level validation of attention-ranked residues.

## 2 Methods

### 2.1 Problem definition

The Drug-Target Interaction (DTI) prediction task can be formally defined as a binary classification problem: given a drug compound and a target protein, the objective is to determine whether an interaction exists between them. In this study, a drug is co-represented by its two-dimensional (2D) molecular graph *G* and its corresponding SMILES sequence *S* (Weininger, 1988; Weininger et al., 1989). A target protein is represented by its amino acid sequence $P=\left(a_{1},...,a_{n}\right)$ , where $a_{i}$ denotes the i amino acid residue in the sequence.

The molecular graph and SMILES sequence provide complementary views of the same compound. The molecular graph encodes atom connectivity and topological structure, whereas the SMILES sequence can be treated as chemical language and encoded to capture contextual patterns related to chemical fragments and functional groups (Krenn et al., 2022; Kearnes et al., 2016; Chithrananda et al., 2022; Liu et al., 2025). Each modality has limitations when used alone. SMILES strings may not fully represent three-dimensional molecular conformations, while graph representations may not capture all contextual chemical patterns expressed in sequential notation. Therefore, this study focuses on integrating graph-based and SMILES-based drug representations and using the fused drug features for DTI prediction.

Given a protein sequence *P*, a drug’s molecular graph *G*, and its SMILES sequence *S*, DTI prediction aims to learn an end-to-end model *M* that efficiently extracts and fuses fine-grained drug features from these multi-modal inputs. The model then learns drug-protein interaction patterns and maps the resulting joint representation to a probability score $p\in \left[0,1\right]$ .

### 2.2 Model architecture

**FIGURE 1:**
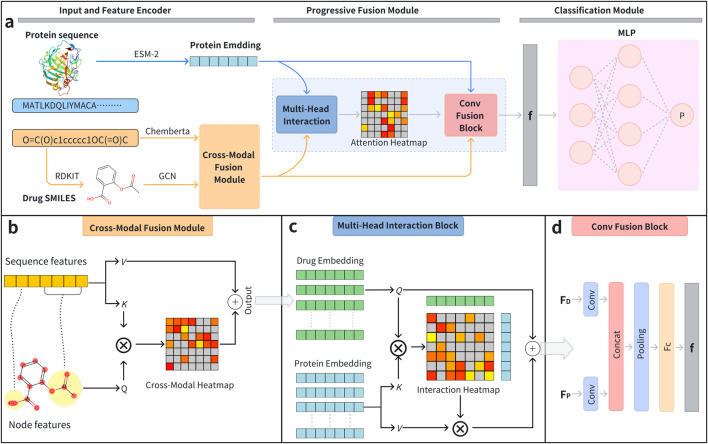
Architecture of the CMA-DTI framework. **(a)** Overall schematic. Drug molecules and proteins are encoded using GCN, ChemBERTa, and ESM-2, respectively. The drug representations are integrated by the intra-drug cross-modal fusion module, and the fused drug representation is combined with protein residue embeddings by the drug–protein interaction module. The resulting features are aggregated and passed to a fully connected classifier to predict the DTI probability. **(b)** Cross-modal fusion module. This module integrates GCN node representations with ChemBERTa token embeddings using cross-attention, producing context-aware drug node representations. **(c)** Drug–protein interaction module. This module uses multi-head attention to compute relevance patterns between fused drug node representations and ESM-2 residue embeddings. **(d)** Convolutional fusion module. This module applies 1D convolutional layers to the interaction features and generates the joint representation for final prediction.

CMA-DTI is a cross-modal fusion and attentive interaction framework for DTI prediction. As illustrated in Figure 1a, the architecture consists of four modules: (i) a feature encoder, (ii) an intra-drug cross-modal fusion module, (iii) a drug–protein interaction module, and (iv) a prediction module.

First, the feature encoder processes the drug and protein inputs in parallel. A Graph Convolutional Network is used to encode the molecular graph and generate atom-level node representations. ChemBERTa is used to encode the SMILES sequence and produce token-level chemical language representations. For the target protein, ESM-2 is used to encode the amino acid sequence and obtain residue-level embeddings.

Second, the intra-drug cross-modal fusion module receives the GCN-based node representations and the ChemBERTa-derived token representations. Through cross-attention, this module models soft relevance patterns between graph nodes and SMILES tokens and generates fused drug node representations.

Third, the fused drug node representations and ESM-2 residue embeddings are passed to the drug–protein interaction module. This module uses multi-head attention to model local relevance patterns between drug nodes and protein residues, producing an interaction-aware feature sequence.

Finally, the prediction module aggregates the interaction-aware features through pooling and a multilayer perceptron, and outputs the predicted interaction probability. The convolutional fusion block used to process the interaction-aware features before final prediction is shown in Figure 1d. The attention weights generated by the intra-drug and drug–protein attention modules are used for attention-based interpretation, while the final prediction is optimized through supervised DTI classification.

#### 2.2.1 Protein Representation Module

The Protein Representation Module extracts contextual feature representations from protein amino acid sequences for subsequent drug–protein interaction modeling. To this end, we use the pre-trained protein language model ESM-2 (esm2_t33_650M_UR50D) trained by Meta AI. We use the Hugging Face Transformers library (Wolf et al., 2020) to load the pre-trained ESM-2 model and its corresponding tokenizer.

Given an input protein sequence $P=\left(a_{1},...,a_{n}\right)$ , the tokenizer first converts it into a sequence of tokens $\left(t_{1},...,t_{L_{p}}\right)$ . This sequence is then padded to accommodate variable sequence lengths within a batch, and a corresponding attention mask, $M_{protein}$ , is generated to distinguish real tokens from padding tokens. The token sequence and the attention mask are then fed into the pre-trained ESM-2 model.

The ESM-2 model processes the token sequence via its multi-layer Transformer architecture, the core mechanism of which is Multi-Head Self-Attention. For each layer l , the computation of the self-attention layer can be summarized as the main mathematical formulations used in the model are provided in Equations 1–13, covering protein self-attention, molecular graph encoding, graph–SMILES fusion, drug–protein attention, pooling, prediction, and loss computation.

$$Q_{SA}^{\left(l\right)}=H^{\left(l\right)}W_{Q}^{\left(l\right)},K_{SA}^{\left(l\right)}=H^{\left(l\right)}W_{K}^{\left(l\right)},V_{SA}^{\left(l\right)}=H^{\left(l\right)}W_{V}^{\left(l\right)}$$ (1)

$${\text{Attention}}_{SA}^{\left(l\right)}=\text{Softmax}\left(\frac{Q_{SA}^{\left(l\right)}{\left(K_{SA}^{\left(l\right)}\right)}^{T}}{\sqrt{d_{model}/H}}+M_{protein}\right)$$ (2)

$$H^{\left(l+1\right)}=\text{Concat}\left({\text{head}}_{1},...,{\text{head}}_{H}\right)W_{O}^{\left(l\right)}$$ (3)

where $H^{\left(l\right)}$ represents the input feature sequence to layer l (initially the token embeddings); $W_{Q}^{\left(l\right)}$ , $W_{K}^{\left(l\right)}$ , $W_{V}^{\left(l\right)}$ , $W_{O}^{\left(l\right)}$ are learnable weight matrices; H is the number of attention heads; and $d_{model}$ is the model’s hidden dimension.

After processing the token sequence through the multi-layer Transformer architecture, ESM-2 outputs contextual residue-level representations. We extract the final hidden state, $H_{protein}\in R^{L_{p}\times D_{protein}}$ , from the model’s last layer to serve as the feature representation for each amino acid residue. This results in a protein residue feature sequence, $H_{protein}$ , which, along with the corresponding attention mask $M_{protein}$ , is subsequently used for attention computations within the Drug-Protein Interaction Module. Compared with fixed embeddings or simple sequence descriptors, ESM-2 provides contextual residue-level representations that capture both local and long-range sequence information.

#### 2.2.2 Drug Representation Module

The Drug Representation Module encodes both molecular graph structure and SMILES-based chemical sequence information to generate fused drug representations for subsequent interaction modeling. It comprises a Graph Convolutional Network (GCN), a pre-trained chemical language model (ChemBERTa), and an Intra-drug Cross-modal Fusion Module. Specifically, the Intra-drug Cross-modal Fusion Module models soft relevance between graph-based node representations and SMILES-derived token representations, generating fused drug node representations for subsequent interaction modeling.

Molecular graphs represent atoms and bonds and encode topological information such as neighborhood relationships (Tsubaki et al., 2019). First, we represent the drug structure as a two-dimensional (2D) molecular graph $G=\left(V,E\right)$ , where V is the set of atoms (nodes) and E is the set of chemical bonds (edges). Each SMILES string is converted into a DGL graph object using functionalities provided in the DGL-LifeSci library (Li et al., 2021). For each node $v_{i}\in V$ in the graph, we initialize its feature vector $h_{v_{i}}^{\left(0\right)}$ based on its chemical properties. Following the implementation in DGL-LifeSci, each atom is described by a 74-dimensional feature vector encoding eight key properties: atom type, atomic degree, number of implicit hydrogens, formal charge, number of radical electrons, atomic hybridization, total number of hydrogens, and whether the atom is aromatic.

Following prior work (Wang et al., 2021), we use a Graph Convolutional Network (GCN) to encode the drug graph. The GCN processes these atomic node features and the graph’s connectivity. A GCN layer updates node features by aggregating neighborhood information, and its general form can be expressed as:

$$h_{v_{i}}^{\left(l+1\right)}=\sigma \left(\sum_{u\in N\left(v_{i}\right)}\;\frac{1}{c_{v_{i},u}}W^{\left(l\right)}h_{u}^{\left(l\right)}+b^{\left(l\right)}\right)$$ (4)

Where $h_{u}^{\left(l\right)}$ is the feature vector of node u at layer l ; $N\left(v_{i}\right)$ is the set of neighbors of node $v_{i}$ ; $c_{v_{i},u}$ is a normalization constant; $W^{\left(l\right)}$ is a learnable weight matrix; $b^{\left(l\right)}$ is a bias term; and σ is an activation function. After stacking multiple GCN layers, we capture atom-level (node-level) structural features and connectivity information, yielding a drug node feature sequence $H_{GCN}\in R^{N\times D_{GCN}}$ , where N is the number of drug nodes (including padding) and $D_{GCN}$ is the GCN’s output feature dimension. To handle drug molecules of varying sizes and enable efficient batch processing, we set a maximum allowable number of nodes when constructing the DGL graphs and apply corresponding padding.

Second, we use the drug SMILES sequence, $S=\left(t_{1},...,t_{L_{c}}\right)$ , as another representational modality, where $t_{j}$ represents the j -th token in the SMILES string. We encode the SMILES sequence using ChemBERTa, a chemical language model pre-trained on SMILES strings. The ChemBERTa model processes the token sequence through its Transformer architecture, outputting a sequence of drug token-level feature representations, $H_{ChemBERTa}\in R^{L_{c}\times D_{ChemBERTa}}$ , where $L_{c}$ is the number of tokens (including padding) and $D_{ChemBERTa}$ is ChemBERTa’s output feature dimension. ChemBERTa provides contextual token representations that encode chemical language patterns and functional group-related information from SMILES strings.

Subsequently, to integrate these two complementary modalities, we feed the drug node feature sequence $H_{GCN}$ and the ChemBERTa token feature sequence $H_{ChemBERTa}$ into the Intra-drug Cross-modal Fusion Module (as shown in Figure 1b). The core of this module is a cross-attention mechanism. It first projects $H_{GCN}$ into the same feature space as $H_{ChemBERTa}$ to serve as the Query: $Q_{drug_{\_}\mathrm{int}}=H_{GCN}W_{Q_{\_}\mathrm{int}}$ . It then uses $H_{ChemBERTa}$ as both the Key, $K_{drug_{\_}\mathrm{int}}$ , and the Value, $V_{drug_{\_}\mathrm{int}}$ . The cross-attention weights are computed as follows:

$$A_{drug_{\_}\mathrm{int}}=\text{Softmax}\left(\frac{Q_{drug_{\_}\mathrm{int}}K_{drug_{\_}\mathrm{int}}^{T}}{\sqrt{D_{ChemBERTa}}}\right)$$ (5)

where $A_{drug_{i}nt}\in R^{N\times L_{c}}$ is the attention weight matrix, with each element $a_{i,j}$ representing the attention paid by the i -th drug node to the j -th ChemBERTa token. Through these weights, the module models soft relevance between drug graph nodes and ChemBERTa-derived SMILES tokens. Finally, the fused drug node feature sequence, $H_{fused_{\_}nodes}\in R^{N\times D_{ChemBERTa}}$ , is generated by computing a weighted sum of the Values:

$$H_{fused_{\_}nodes}=A_{drug_{\_}\mathrm{int}}V_{drug_{\_}\mathrm{int}}$$ (6)

This fused node feature sequence is used as the drug representation for subsequent drug–protein interaction modeling.

It should be noted that CMA-DTI does not assume a deterministic one-to-one correspondence between molecular graph atoms and SMILES tokens. In the intra-drug cross-attention module, graph node embeddings generated by the GCN are used as queries, whereas ChemBERTa token embeddings derived from the SMILES sequence are used as keys and values. Therefore, the resulting attention weights represent a soft contextual relevance distribution from each graph node to SMILES-derived chemical language tokens, rather than a hard atom-token alignment.

This design accommodates the mismatch between graph atoms and SMILES tokens. For example, SMILES strings may contain branch symbols, ring-closure indices, aromatic notation, stereochemical symbols, multi-character atom tokens, and tokenizer-specific special tokens. These tokens are not forced to correspond to individual atoms. Instead, they contribute to the fused graph-node representation according to their learned contextual relevance. In this way, the intra-drug cross-attention module provides a soft graph–SMILES relevance modeling mechanism while avoiding the need for manually defined atom-token matching rules.

#### 2.2.3 Feature interaction and output module

The Feature Interaction and Output Module integrates drug and protein representations and outputs the predicted interaction probability. To model local drug–protein relevance patterns, this module applies a Drug–Protein Multi-Head Attention network to fused drug nodes and protein residue embeddings. This module consists of a core Drug-Protein Interaction Module, followed by a pooling layer and a fully-connected classification layer. The learned attention weights can be visualized to provide attention-based relevance patterns for interpretation.

The Drug-Protein Interaction Module (as depicted in Figure 1c) receives the fused drug node feature sequence, $H_{fused_{-}nodes}\in R^{N\times D_{drug_{\_}fused}}$ , from the Drug Representation Module, and the protein residue feature sequence, $H_{protein}\in R^{L_{p}\times D_{protein}}$ , from the Protein Representation Module. Here, N is the number of drug nodes (including padding), $L_{p}$ is the number of protein residues (including padding), $D_{drug_{\_}fused}$ is the dimension of the fused drug node features, and $D_{protein}$ is the dimension of the protein features.

The core of this module is a Multi-Head Attention network. To ensure dimensional consistency between the drug and protein features for the attention computation, the fused drug node feature sequence $H_{fused_{\_}nodes}$ is first transformed into the Query $Q\in R^{N\times D_{protein}}$ via a linear projection layer $W_{Q}\in R^{D_{drug_{f}used}\times D_{protein}}$ :

$$Q=H_{fused_{\_}nodes}W_{Q}+b_{Q}$$ (7)

Here, **Q** serves as the Query, while $H_{protein}$ serves as both the Key, **K**, and the Value, **V**. The Multi-Head Attention mechanism projects the inputs into H different subspaces for parallel computation. Each head h has its own projection matrices $W_{Q}^{\left(h\right)}$ , $W_{K}^{\left(h\right)}$ , $W_{V}^{\left(h\right)}$ . For each head h , the attention weights $A^{\left(h\right)}\in R^{N\times L_{p}}$ are computed as follows:

$$A^{\left(h\right)}=\text{Softmax}\left(\frac{\left(QW_{Q}^{\left(h\right)}\right){\left(H_{protein}W_{K}^{\left(h\right)}\right)}^{T}}{\sqrt{d_{k}}}\right)$$ (8)

where $d_{k}=D_{protein}/H$ is the feature dimension per head. The Softmax function is applied along the protein residue dimension (i.e., the $L_{p}$ dimension), ensuring that the attention weights from each drug node to all protein residues sum to 1. An attention mask is applied during this computation to ignore padded positions.

Through these attention weights, the module models local relevance patterns between drug nodes and protein residues. The output of each head, $O^{\left(h\right)}\in R^{N\times d_{v}}\left(d_{v}=D_{protein}/H\right)$ , is obtained by applying the attention weights to the Values:

$$O^{\left(h\right)}=A^{\left(h\right)}\left(H_{protein}W_{V}^{\left(h\right)}\right)$$ (9)

Finally, the outputs of all heads are concatenated along the feature dimension and then passed through a final linear projection with weights $W_{O}\in R^{D_{protein}\times D_{protein}}$ to produce the module’s output sequence, $H_{interact_{s}eq}\in R^{N\times D_{protein}}$ :

$$H_{interact\_seq}=\text{Concat}\left(O^{\left(1\right)},...,O^{\left(H\right)}\right)W_{O}+b_{O}$$ (10)

The module’s output, $H_{interact\_seq}$ , is a sequence whose length corresponds to the number of drug nodes. Each feature vector in this sequence represents the interaction-aware information associated with the corresponding drug node and the protein sequence.

Subsequently, this interaction-aware output sequence, $H_{interact\_seq}$ , is fed into a pooling layer (Masked Mean Pooling), which aggregates it into a fixed-dimension joint feature vector, $z_{joint}\in R^{D_{protein}}$ . This pooling operation accounts for the padding of drug nodes, and its formula can be expressed as:

$$z_{joint}=\text{MaskedMeanPooling}\left(H_{interact_{\_}seq},M_{nodes}\right)$$ (11)

where $M_{nodes}\in R^{N}$ is a binary mask indicating the positions of the actual (non-padded) drug nodes. This joint feature vector summarizes the interaction-aware representation of the drug–protein pair.

Finally, this joint feature vector $z_{joint}$ is fed into a fully-connected classification layer, which maps it to the final prediction score p . This layer, typically composed of multiple linear layers and activation functions, outputs a probability value for the binary classification task through a final Sigmoid function:

$$p=\text{Sigmoid}\left(\text{MLP}\left(z_{joint}\right)\right)$$ (12)

For the loss function, we employ the standard Binary Cross-Entropy (BCE) loss, often referred to as Logloss:

$$L=-\frac{1}{M}\sum_{i=1}^{M}\;\left[y_{i}\log\left(p_{i}\right)+\left(1-y_{i}\right)\log\left(1-p_{i}\right)\right]$$ (13)

## 3 Experiments

### 3.1 Datasets

Two benchmark datasets were used in this study: BindingDB (Gilson et al., 2016) and BioSNAP (Zitnik et al., 2018). The dataset statistics are summarized in Table 2.

**TABLE 2:** Statistics of experimental datasets.

Datasets	Proteins	Drugs	Positives	Negatives	Interactions
BindingDB	1,145	10,433	34,185	34,185	68,370
BioSNAP	2,271	4,526	15,137	15,137	30,274

#### 3.1.1 BindingDB dataset

BindingDB is a public database that contains experimentally measured binding information between small molecules and protein targets. Following common practice in binary DTI prediction, interactions with IC50, Ki, or Kd values of 100 nM or less were treated as positive samples. Negative samples were generated by randomly pairing drugs and targets without annotated interactions, with the number of negative samples matched to the number of positive samples.

After preprocessing and balancing, the BindingDB dataset used in this study contained 1,145 proteins, 10,433 drugs, 34,185 positive interactions, and 34,185 negative interactions, resulting in 68,370 drug–target pairs.

#### 3.1.2 BioSNAP dataset

BioSNAP provides curated biomedical network data, including known drug–target interactions. We used the processed BioSNAP DTI dataset and generated negative samples by randomly pairing drugs and targets without annotated interactions, matching the number of positive samples.

After preprocessing and balancing, the BioSNAP dataset used in this study contained 2,271 proteins, 4,526 drugs, 15,137 positive interactions, and 15,137 negative interactions, resulting in 30,274 drug–target pairs.

### 3.2 Baseline methods

To evaluate CMA-DTI, we compared it with eight representative baseline methods, including two conventional machine learning methods and six deep learning-based DTI models.

First, we included two conventional machine learning baselines: Support Vector Machine (SVM) (Cortes and Vapnik, 1995) and Random Forest (RF) (Ho, 1995). Both methods used concatenated ECFP and PSC fingerprints (Cao et al., 2013) as input features, representing descriptor-based DTI prediction approaches.

Second, we compared CMA-DTI with six deep learning-based DTI models. DeepConv-DTI (Lee et al., 2019) uses convolutional neural networks to extract protein sequence features and fully connected layers to encode ECFP4 drug fingerprints. GraphDTA (Nguyen et al., 2021) encodes drug molecular graphs using graph neural networks and protein sequences using convolutional neural networks; to adapt it to binary classification, we added a sigmoid output layer and optimized it with cross-entropy loss. MolTrans (Huang et al., 2021) uses Transformer-based representations and an interaction module to model drug–protein substructure relationships. DrugBAN (Bai et al., 2023) encodes drug molecular graphs and protein sequences and applies a bilinear attention network to generate joint drug–target representations. MGMA-DTI (Li et al., 2025) uses multi-order gated convolutions and multi-attention fusion to model drug–target interaction features. TriDTI (Yun et al., 2026) is a recent multimodal DTI framework that integrates structural, sequential, and relational features with cross-modal alignment and attention-based fusion.

Together, these baselines cover descriptor-based machine learning, CNN-based models, graph-based models, Transformer-based models, bilinear attention models, and recent multimodal DTI approaches.

### 3.3 Experimental setup, implementation details, and evaluation metrics

For the main benchmark comparison under random pair splits, we employed a 10-fold cross-validation strategy. Specifically, each dataset was partitioned into 10 mutually exclusive folds. In each run, one fold was used as the test set, while the remaining folds were used for model training and validation. A fixed portion of the training data was further set aside as the validation set for model selection and early stopping. The results of the main benchmark comparison are reported as the mean and standard deviation across the 10 folds.

To further evaluate cold-start generalization, we additionally constructed fixed cold-drug and cold-target splits for BindingDB and BioSNAP. In the cold-drug split, drugs in the validation and test sets were excluded from the training set. In the cold-target split, protein targets in the validation and test sets were excluded from the training set. These splits were used as stricter evaluations of generalization to unseen drugs or unseen protein targets. Since these cold-start experiments were conducted using fixed train/validation/test partitions, their results are reported as single-run test performance rather than 10-fold averages.

The proposed CMA-DTI framework was implemented using PyTorch and trained on an NVIDIA A6000 GPU. Model parameters were optimized using the Adam optimizer. The maximum number of training epochs was set to 100, and early stopping was applied according to validation performance. The model checkpoint with the best validation AUROC was selected for final testing.

For model configuration, the initial atom feature dimension of the molecular graph was set to 74, and the maximum number of drug nodes was padded to 290 for batch processing. For sequence-based representations, ChemBERTa-77M-MLM was used to encode drug SMILES strings, producing 768-dimensional token embeddings, and ESM-2 (esm2_t33_650M_UR50D) was used to encode protein sequences with a maximum sequence length of 512. The hidden dimension of the final multilayer perceptron decoder was set to 512.

Hyperparameters were selected through grid search on the validation set. The searched hyperparameters included the learning rate, batch size, number of GNN layers, number of attention heads in the intra-drug and drug–protein attention modules, and attention dropout rate. The search ranges and final selected values are summarized in Table 3.

**TABLE 3:** Grid search range and final selected values of main hyperparameters for CMA-DTI.

Hyperparameter	Search range	Selected value
Learning rate	{1e-5, 5e-5, 1e-4, 5e-4}	5e-5
Batch size	{32, 64, 128}	64
Number of GNN layers	{2, 3, 4}	3
Number of attention heads	{2, 4, 8}	2
Attention dropout rate	{0.1, 0.2, 0.3}	0.2

**FIGURE 2:**
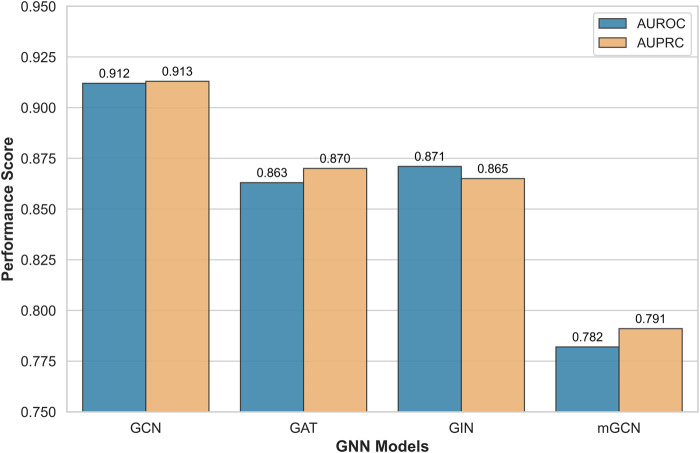
Comparison of GCN, GAT, GIN, and mGCN as drug graph encoders on the BioSNAP dataset. The bars show AUROC and AUPRC values obtained while keeping the remaining CMA-DTI modules unchanged.

We also compared four graph neural network architectures, including GCN, GAT, GIN, and a modified GCN variant (mGCN), as the drug graph encoder on the BioSNAP dataset while keeping the remaining modules unchanged. As shown in Figure 2, none of the tested alternatives clearly outperformed GCN in terms of AUROC or AUPRC. Considering its comparable predictive performance and lower computational cost, GCN was selected as the default graph encoder in CMA-DTI.

To evaluate classification performance, we used AUROC and AUPRC as the primary threshold-independent metrics. We also reported threshold-dependent metrics, including Accuracy, Sensitivity, Specificity, Precision, and F1-score. Sensitivity was defined as TP/(TP + FN), and Specificity was defined as TN/(TN + FP). For threshold-dependent metrics, the decision threshold was selected using the validation set and then applied to the test set.

### 3.4 Results

To evaluate CMA-DTI, we compared it with traditional machine learning methods and representative deep learning baselines on BindingDB and BioSNAP. As shown in Table 4, CMA-DTI achieved competitive performance on both datasets. Compared with RF and SVM, CMA-DTI showed improved predictive performance, suggesting the benefit of learned drug and protein representations. Compared with the deep learning baselines, CMA-DTI also achieved higher overall performance, indicating that graph-based drug features, chemical language representations, protein language model embeddings, and attention-based interaction modeling jointly contribute to DTI prediction.

**TABLE 4:** Performance comparison on the BindingDB and BioSNAP datasets, averaged over 10-fold cross-validation.

Dataset	Method	AUROC	AUPRC	Accuracy	Sensitivity	Specificity
BindingDB	SVM	0.939 ± 0.001	0.928 ± 0.002	0.825 ± 0.004	0.781 ± 0.014	0.886 ± 0.012
	RF	0.942 ± 0.011	0.921 ± 0.016	0.880 ± 0.012	0.875 ± 0.023	0.892 ± 0.020
	DeepConv-DTI	0.945 ± 0.002	0.925 ± 0.005	0.882 ± 0.007	0.873 ± 0.018	0.894 ± 0.009
	GraphDTA	0.951 ± 0.002	0.934 ± 0.002	0.888 ± 0.005	0.882 ± 0.012	0.897 ± 0.008
	MolTrans	0.952 ± 0.002	0.936 ± 0.001	0.887 ± 0.006	0.877 ± 0.016	0.902 ± 0.009
	DrugBAN	0.960 ± 0.001	0.947 ± 0.002	0.904 ± 0.004	0.900 ± 0.008	0.908 ± 0.004
	MGMA-DTI	0.958 ± 0.002	0.943 ± 0.002	0.908 ± 0.004	0.902 ± 0.008	0.914 ± 0.003
	TriDTI	0.957 ± 0.003	0.947 ± 0.002	0.910 ± 0.006	**0.908 ± 0.006**	0.913 ± 0.004
	CMA-DTI	**0.962 ± 0.001**	**0.948 ± 0.002**	**0.913 ± 0.006**	0.906 ± 0.008	**0.921 ± 0.005**
BioSNAP	SVM	0.862 ± 0.007	0.864 ± 0.004	0.777 ± 0.011	0.711 ± 0.042	0.841 ± 0.028
	RF	0.860 ± 0.005	0.886 ± 0.005	0.804 ± 0.005	0.823 ± 0.032	0.786 ± 0.025
	DeepConv-DTI	0.886 ± 0.006	0.890 ± 0.006	0.805 ± 0.009	0.760 ± 0.029	0.851 ± 0.013
	GraphDTA	0.887 ± 0.008	0.890 ± 0.007	0.800 ± 0.007	0.745 ± 0.032	**0.854 ± 0.025**
	MolTrans	0.895 ± 0.004	0.897 ± 0.005	0.825 ± 0.010	0.818 ± 0.031	0.831 ± 0.013
	DrugBAN	0.903 ± 0.005	0.902 ± 0.004	0.834 ± 0.008	0.820 ± 0.021	0.847 ± 0.010
	MGMA-DTI	0.909 ± 0.001	0.911 ± 0.002	0.844 ± 0.004	0.843 ± 0.008	0.844 ± 0.004
	TriDTI	0.907 ± 0.001	0.909 ± 0.002	0.845 ± 0.004	0.847 ± 0.008	0.843 ± 0.004
	CMA-DTI	**0.912 ± 0.005**	**0.913 ± 0.006**	**0.851 ± 0.009**	**0.859 ± 0.028**	0.849 ± 0.023

The results are presented as mean ± standard deviation. The best result for each dataset and metric is marked in bold and the second-best result is underlined.

To assess cold-start generalization, we further evaluated CMA-DTI under cold-drug and cold-target splits. In the cold-drug setting, drugs in the validation and test sets were excluded from training. In the cold-target setting, protein targets in the validation and test sets were excluded from training. These settings provide stricter evaluations than random pair splits.

As shown in Table 5, CMA-DTI maintained predictive performance under both cold-start settings. On BindingDB, AUROC/AUPRC reached 0.938/0.915 under the cold-drug split and 0.908/0.885 under the cold-target split. On BioSNAP, the corresponding AUROC/AUPRC values were 0.872/0.881 and 0.857/0.869, respectively. These results indicate that CMA-DTI retained predictive ability when evaluated on unseen drugs and unseen protein targets.

**TABLE 5:** Generalization performance of CMA-DTI under cold-drug and cold-target splits.

Dataset	Split	AUROC	AUPRC	Sensitivity	Specificity	Accuracy	F1
BindingDB	cold-drug	0.938	0.915	0.867	0.881	0.873	0.875
BindingDB	cold-target	0.908	0.885	0.841	0.863	0.852	0.828
BioSNAP	cold-drug	0.872	0.881	0.790	0.809	0.800	0.801
BioSNAP	cold-target	0.857	0.869	0.783	0.786	0.784	0.785

### 3.5 Ablation study

To evaluate the contribution of the main components in CMA-DTI, we conducted ablation experiments on the BindingDB dataset using the same setting as the main comparison. Four component-level variants were considered. In the **w/o ESM-2 (CNN)** variant, the ESM-2 protein encoder was replaced with a three-layer 1D-CNN. In the **w/o Multi-modal Drug Representation (ECFP)** variant, the GCN, ChemBERTa encoder, and intra-drug fusion module were replaced with 2048-bit ECFP fingerprints. In the **w/o Internal Cross-Attention (Concat)** variant, the intra-drug cross-attention module was replaced with simple feature concatenation. In the **w/o Drug–Protein Attention (Global Concat)** variant, the local drug–protein interaction module was replaced with global pooling and concatenation.

The component-level ablation results are summarized in Table 6. The full CMA-DTI model achieved the best overall performance among the compared variants, with AUROC and AUPRC values of 0.962 and 0.946, respectively. Replacing the multimodal drug encoder with ECFP fingerprints led to a clear performance decrease, indicating that learned graph- and language-based drug representations provide useful information beyond predefined molecular descriptors. Removing the drug–protein attention module also reduced overall performance, particularly in specificity, suggesting that local interaction modeling helps reduce false-positive predictions. Replacing intra-drug cross-attention with simple concatenation also decreased AUROC and AUPRC, indicating that graph–SMILES fusion contributes to predictive performance.

**TABLE 6:** Results of the ablation study on BindingDB.

Model variant	AUROC	AUPRC	Sensitivity	Specificity	Accuracy
CMA-DTI	**0.962**	**0.946**	0.905	**0.921**	**0.913**
No ESM-2	0.956	0.940	0.898	0.905	0.902
No Adv. Drug Enc	0.947	0.926	**0.921**	0.851	0.880
No Cross-Attention	0.948	0.932	0.876	0.901	0.887
No D-P Attention	0.955	0.933	0.918	0.889	0.891

The best result for each metric is marked in bold and the second-best result is underlined.

To further compare different intra-drug fusion strategies, we evaluated cross-attention against simple concatenation, element-wise addition, and gated fusion on BindingDB. In the addition variant, graph-based and SMILES-derived representations were projected into the same latent space and combined by element-wise addition. In the gated fusion variant, a learnable gate was used to adaptively balance the two modalities. All other modules and hyperparameters were kept unchanged.

As shown in Table 7, gated fusion outperformed addition and concatenation, suggesting that adaptive multimodal fusion was more effective than fixed feature combination in this setting. CMA-DTI achieved the highest values across all reported metrics. Compared with gated fusion, intra-drug cross-attention improved AUROC from 0.956 to 0.962 and AUPRC from 0.940 to 0.946. These results suggest that modeling soft graph–SMILES relevance through cross-attention provides additional benefit over standard fusion strategies. The cross-attention module also produces atom/token-level relevance patterns that can support attention-based interpretation.

**TABLE 7:** Fusion-strategy ablation of the intra-drug cross-modal fusion module on BindingDB.

Model variant	AUROC	AUPRC	Sensitivity	Specificity	Accuracy
CMA-DTI	**0.962**	**0.946**	**0.905**	**0.921**	**0.913**
Gated fusion	0.956	0.940	0.898	0.916	0.907
Addition fusion	0.952	0.936	0.889	0.909	0.899
Concat fusion	0.948	0.932	0.876	0.901	0.887

Cross-attention, gated fusion, addition fusion, and concatenation refer to different strategies for integrating GCN-based graph representations and ChemBERTa-derived SMILES representations. Bold values indicate the best performance for each metric.

### 3.6 Case study

To examine whether the attention patterns generated by CMA-DTI are consistent with known structural interaction regions, we analyzed three representative drug–target complexes with experimentally resolved PDB structures: 8V5H (Gallego et al., 2024), 6Q13 (Rai et al., 2020), and 7T4I (Huang et al., 2023). These cases were used as external structural examples to qualitatively inspect whether high-attention atoms or residues were located near ligand-binding regions.

#### 3.6.1 Qualitative structural analysis of attention patterns

**FIGURE 3:**
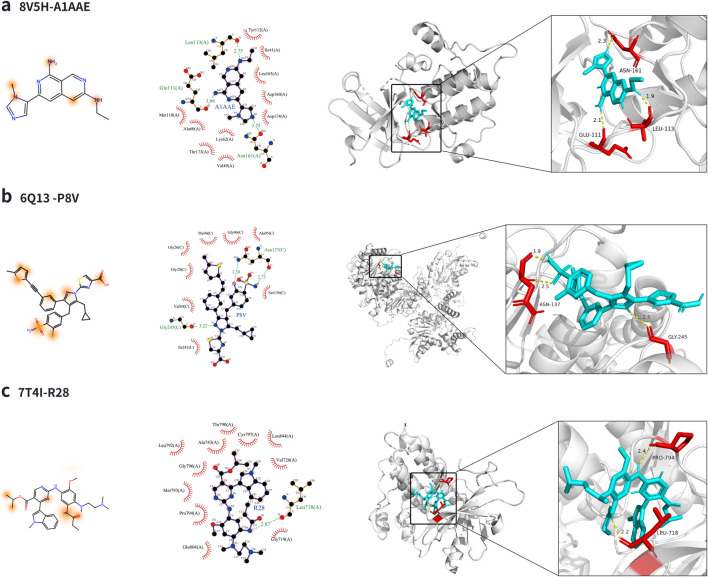
Visualization of ligand attention patterns and binding-pocket regions in representative structural cases. Panel **(a)** shows the 8V5H-A1AAE complex, panel **(b)** shows the 6Q13-P8V complex, and panel **(c)** shows the 7T4I-R28 complex. The left side of each panel shows the 2D ligand structure, with high-attention atoms highlighted in orange. The middle panel shows ligand–protein interaction diagrams generated from the corresponding crystal structures using LigPlot+ (Laskowski and Swindells, 2011). The right side shows the 3D ligand-binding pocket, with attention-ranked residues located near the ligand-binding region highlighted in red and the ligand shown in cyan. The remaining residues, secondary structure elements, and surface maps are shown in gray. The 2D ligand structures were visualized using RDKit (Landrum, 2006), and the 3D structures were visualized using PyMOL (Schrödinger and DeLano, 2020).

As shown in Figure 3, CMA-DTI highlighted several ligand atoms located near experimentally observed interaction regions. In the 8V5H complex, atoms within the amino and imino groups received relatively high attention scores and were located near hydrogen-bonding residues such as Glu-111 and Leu-113. In the 6Q13 complex, attention was assigned to the sulfonamide and carboxylic acid regions, which are positioned near residues such as Asn-137, Arg-168, His-192, and Tyr-144. In the 7T4I complex, atoms near pharmacophoric regions of Mobocertinib were highlighted, although some attention signals were also assigned to atoms without direct annotated interactions. These observations suggest that the attention maps can reflect chemically relevant regions in selected cases, but should not be interpreted as exact binding-site annotations.

#### 3.6.2 Dual-attention analysis of the 6Q13-P8V complex

**FIGURE 4:**
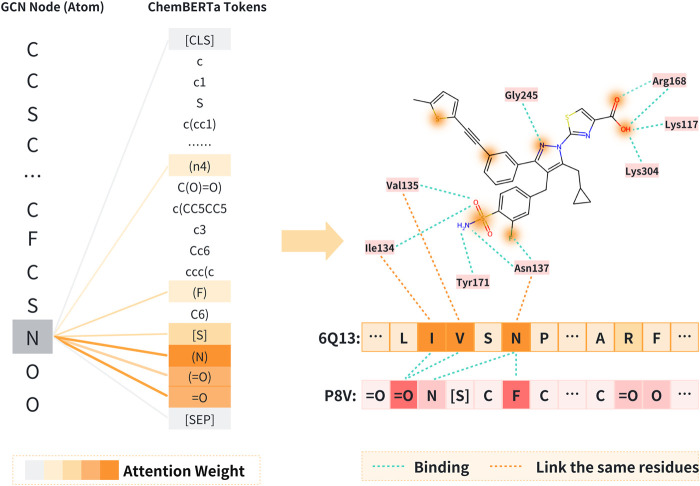
Attention maps for the 6Q13-P8V pair. The figure shows the intra-drug cross-modal attention map linking graph nodes to SMILES tokens and the drug–protein attention map linking drug atoms to protein residues. These maps are used to visualize attention-based relevance patterns rather than causal interaction mechanisms.

Using the 6Q13-P8V complex as a representative example, we examined the two attention layers in CMA-DTI (Figure 4). The intra-drug cross-attention module associated graph-based atom representations with ChemBERTa-derived SMILES token representations. For example, the nitrogen atom in the molecular graph showed attention to tokens related to the sulfonamide fragment. The subsequent drug–protein attention module assigned relevance between the fused ligand representation and residues near the binding pocket, including Asn-137. This two-level attention pattern provides a traceable relevance path from intra-drug representation fusion to drug–protein interaction modeling. However, these attention weights should be viewed as relevance indicators rather than causal mechanistic explanations.

To quantify whether attention-ranked residues were enriched in experimentally observed ligand-binding regions, we performed pocket-level validation on the representative 6Q13-P8V case. Binding-pocket residues were defined as protein residues with at least one heavy atom within 5 Å of the co-crystallized ligand. Residue-level attention scores were obtained by averaging attention weights across attention heads and taking the maximum over valid drug atoms.

As shown in Table 8, the top-20, top-30, and top-50 attention-ranked residues recovered 3, 5, and 6 pocket residues, respectively. The corresponding enrichment factors were 2.257, 2.508, and 1.805, all higher than the random residue-ranking baselines. Although the absolute Precision@k and Recall@k values were modest, the enrichment over random rankings suggests that high-attention residues were moderately associated with the structure-derived binding pocket in this representative case.

**TABLE 8:** Pocket-level validation of attention-ranked residues in 6Q13-P8V.

Top-k	Recovered	Precision	Recall	F1	EF	Random EF
20	3	0.150	0.136	0.143	2.257	0.964 ± 0.785
30	5	0.167	0.227	0.192	2.508	1.033 ± 0.668
50	6	0.120	0.273	0.167	1.805	0.977 ± 0.479

Residue-level attention scores were calculated using mean-head/max-atom aggregation. Recovered denotes the number of pocket residues among the top-k attention-ranked residues. Binding-pocket residues were defined using a 5 Å cutoff from the co-crystallized ligand. Random EF, values were calculated from 1,000 random residue rankings.

## 4 Discussion

CMA-DTI was designed to integrate complementary molecular and protein representations for DTI prediction. The results suggest that combining GCN-based molecular graph features, ChemBERTa-derived chemical language representations, and ESM-2 protein residue embeddings is beneficial for predictive performance. The fusion-strategy ablation further shows that intra-drug cross-attention achieved higher performance than concatenation, addition, and gated fusion for integrating graph-based and SMILES-based drug representations. This suggests that modeling soft graph–SMILES relevance patterns can provide useful information beyond standard feature fusion.

The cold-drug and cold-target experiments provide a stricter evaluation of generalization than random pair splits. Although performance decreased under these settings, CMA-DTI retained predictive ability on unseen drugs and unseen protein targets. This suggests that the model does not rely only on pair-level overlap in random splits. At the same time, cold-start prediction remains more challenging than random-split evaluation, especially when the test set contains unseen targets.

The attention analyses provide additional information about the model’s relevance patterns. The qualitative structural cases and the quantitative pocket-level validation on the 6Q13-P8V complex showed that attention-ranked residues can be moderately enriched in structure-derived ligand-binding regions. However, these results should be interpreted as hypothesis-generating evidence rather than causal mechanistic explanations. Because CMA-DTI uses molecular graphs, SMILES strings, and protein sequences as input, it does not explicitly model three-dimensional conformational dynamics.

This study also has limitations related to data curation. Following common practice in binary DTI prediction, positive samples were defined using a 100 nM threshold across IC50, Ki, and Kd values. However, these measurements are not equivalent, and combining potency and affinity values may introduce label noise (Landrum and Riniker, 2024). In addition, negative samples were generated from unannotated drug–target pairs, which may contain unknown interactions. Future work will focus on more strictly curated binding data, broader transfer and cold-start evaluation across independently curated datasets, controlled label-noise sensitivity analysis, and the integration of explicit three-dimensional structural information.

## 5 Conclusion

In this study, we presented CMA-DTI, a cross-modal fusion and attentive interaction framework for drug–target interaction prediction. By integrating GCN-based molecular graph representations, ChemBERTa-derived chemical language representations, and ESM-2 protein residue embeddings, CMA-DTI achieved competitive performance on BindingDB and BioSNAP. Cold-drug and cold-target evaluations suggest that the model retains reasonable predictive ability under stricter unseen-drug and unseen-target settings. The fusion-strategy ablation further supports the contribution of intra-drug cross-attention to graph–SMILES representation fusion. In addition, the qualitative structural cases and quantitative pocket-level validation indicate that the attention patterns can provide hypothesis-generating molecular relevance evidence, although they should not be interpreted as causal mechanistic explanations. Overall, CMA-DTI provides a practical framework for multimodal DTI prediction, and future work will focus on incorporating more strictly curated binding data and explicit three-dimensional structural information to further improve generalization and interpretability.

## Data Availability

The datasets analyzed in this study are publicly available from BindingDB and BioSNAP. The source code used in this study is available at: https://github.com/qinchi1/CMA-DTI. Further inquiries can be directed to the corresponding authors.
